# Was Muller’s 1946 Nobel Prize research for radiation-induced gene mutations peer-reviewed?

**DOI:** 10.1186/s13010-018-0060-5

**Published:** 2018-06-06

**Authors:** Edward J. Calabrese

**Affiliations:** Department of Environmental Health Sciences, School of Public Health and Health Sciences, University of Massachusetts, Morrill I, N344, Amherst, MA 01003 USA

**Keywords:** Nobel prize, Peer-review, Hermann J. Muller, Cancer risk assessment, Mutation

## Abstract

This historical analysis indicates that it is highly unlikely that the Nobel Prize winning research of Hermann J. Muller was peer-reviewed. The published paper of Muller lacked a research methods section, cited no references, and failed to acknowledge and discuss the work of Gager and Blakeslee (PNAS 13:75-79, 1927) that claimed to have induced gene mutation via ionizing radiation six months prior to Muller’s non-data *Science* paper (Muller, Science 66(1699):84-87, 1927a). Despite being well acclimated into the scientific world of peer-review, Muller choose to avoid the peer-review process on his most significant publication. It appears that Muller’s actions were strongly influenced by his desire to claim primacy for the discovery of gene mutation. The actions of Muller have important ethical lessons and implications today, when self-interest trumps one’s obligations to society and the scientific culture that supports the quest for new knowledge and discovery.

## Background

Hermann J. Muller’s reporting that X-rays induced gene mutations in the fruit fly has long been considered an interesting one [8]. Muller was in a tight race with at least three other research groups to be the first to induce gene mutations; the professional stakes were very high [4]. It was believed essential to be the first to report one’s discovery to secure honors, awards and enhanced research support. Muller’s groundbreaking paper was published in the July 22, 1927 issue of *Science* under the title, “The Artificial Transmutation of the Gene” [18]. Since the claimed findings were of fundamental importance, many researchers were extremely disappointed and puzzled that the paper contained no data, only a discussion of data that no one, apparently other than Muller, had seen. To only “discuss” one’s data in this instance was seen as a poorly disguised effort to claim primacy for a novel and significant discovery that was premature. Why the editors at *Science* permitted Muller with their vehicle to manipulate the scientific community, ignoring the standard protocols/rules of scientific publication, has never been clarified by this journal, nor by Muller based on a review of publications and letter correspondence. So bizarre was this deviation from accepted protocol that Muller’s former Ph.D. advisor at Columbia University, Thomas H. Morgan, suggested that perhaps Muller did not have the data to support his claims [8].

## Muller’s Nobel Prize data paper

The rampant speculation came to a head when Muller presented the data some three months after the *Science* publication at the 5th International Genetics Congress (September 11–18, 1927) in Berlin, finally silencing the critics. He showed that X-rays produced transgenerational phenotypic changes in the fruit fly, which he asserted were due to gene mutations. These changes were also induced with striking frequency. Muller became a highly recognized scientific leader within days as the media quickly grasped the potential significance of his discovery. However, he still had not published these research findings. These data, which would provide the basis for his eventual Nobel Prize in 1946, were subsequently published in 1928 in the Proceedings of the International Genetics Congress [20]. Despite its significance, this paper was not widely accessible, being found in few libraries worldwide. Thus, the “data-less/discussion-only” *Science* publication became the publication of note, even today, based on citation frequency. The data-based proceedings paper would be cited only about once/year in the basic Web of Science search until his Nobel Prize in 1946 and even less frequently thereafter.

Nearly a decade ago I obtained the Muller’s 1928 Genetics Congress proceedings paper, being interested to learn more precisely how he verified the 1927 *Science* statements. However, this paper proved to be problematic as it failed to include a methods section, nor a normal discussion section that places his findings within the context of other relevant work, failing to cite any references. This omission was particularly glaring since Gager and Blakeslee [12] had reported in *Proceedings of the National Academy of Sciences* (PNAS) evidence for the first time of true gene mutations (via the use of ionizing radiation), some six months prior to Muller’s *Science* paper. These same researchers also presented their mutation data at the Berlin conference with Muller and are also published in the same International Genetics Congress proceedings. The failure of Muller to acknowledge the January 1927 PNAS Gager and Blakeslee findings/claims is a serious issue, since both groups were claiming primacy for the induction of gene mutation.

Of further relevance was Muller’s failure to address the possibility that his “gene” mutation findings could have been simply due to heritable chromosomal mutation, via the deleting of large gene segments. This issue was a crucial, yet initially hidden, concern of Muller. It was discussed privately by Muller and his colleague/friend Edgar Altenburg during the late summer/fall of 1927. He would eventually publicly discuss the matter at the annual American Association for the Advancement of Science (AAAS) meeting in December 1927 [19] and at a presentation to the National Academy of Sciences (NAS) in the spring of 1928 [21] as he tried to get control of an issue before it might undercut his striking success. However, this issue would not disappear, as Lewis J Stadler, from the University of Missouri, would challenge Muller’s findings starting in 1931 until his death in 1954, claiming he did not induce point mutations but instead induced gapingly large chromosomal deletions [24–26].

In addition to the above issues, Muller’s paper also seemed poorly constructed, requiring revision and clarification to enhance readability. The paper had other limitations of a more specific nature that could have been raised in a review process. While the purpose of the present paper is not to retrospectively review Muller’s paper some 90 years later, but to present a few additional issues that may have warranted responses from contemporary peer-reviewers such as: failure to include a control group for experiment #3, and the use of only two doses, especially when using doses that were very high for experiments #2 and #3 with his new, more sensitive and then unvalidated fruit fly model. Furthermore, the initial experiment (#1) with a standard fruit model was performed with four doses, with the upper doses inducing substantial sterility while the lowest dose, which was still quite high, showed no evidence of transgenerational phenotypic (i.e. mutational) changes. These points were either only casually addressed, or ignored.

Upon reflection, it appeared that there was very little, if any, possibility that this paper had been peer reviewed due to its multiple critical information omissions, experimental design and evaluation questions and a lack of clarity in writing style. Despite its extraordinary gene mutational claims, the manuscript required considerable improvement prior to being acceptable for publication. These issues led to my obtaining a copy of Muller’s research notebook, containing the data for his Nobel Prize research during the time period for the respective experiments. I also obtained numerous communications between Muller and colleagues to explore whether the manuscript was peer reviewed and if it was, what the evaluations were. The information revealed evidence within a letter from Muller to Edgar Altenburg on July 8, 1946 [22] that the manuscript that he read at the 5th International Genetics Congress in Berlin nearly 19 years earlier most likely did not undergo peer-review prior to publication. In that letter, Muller stated:

**“This is to answer your question about when I first suggested the making of cytogenetic maps. In my paper given to the 1927 Congress in Berlin, it is stated on page 245 that the structural changes produced by x-rays should make it possible to obtain more direct evidence of the physical correctness of the linage maps. This paper, though printed first in 1928, was printed from a manuscript given (in) at the time of the Congress in 1927 where it was presented exactly as finally printed.”**

Muller indicates that his presentation at the Congress in 1927 was published exactly as presented with no changes made. This statement reveals that the editor(s) of the Congress very likely published it without peer-review.

## Discussion

A legitimate peer-review may have highlighted important methodological/study design concerns such as the requirement for a methods section, the need to discuss relevant research such as the claims of Gager and Blakeslee [12] for gene mutation findings, as well as addressing whether the high doses of radiation were simply poking large holes via deletions in the chromosomes rather than inducing point mutations. These and other possible clarifications and the further reviewing of a revised manuscript would have delayed publication, possibly threatening Muller’s claim to primacy. The outcome and time-duration of the review process are always difficult to predict. While the letter of July 8, 1946 between Altenburg and Muller as quoted above does not specifically address the issue of peer-review, it nonetheless leads one to reasonably conclude that there was little likelihood of peer review for his Nobel Prize findings.

The failure to receive appropriate peer review created the opportunity for Muller to quickly gain attention and fame, at the expense of others. It also seriously affected the direction of the field since he incorrectly linked the induction of transgenerational phenotypic changes induced by very high doses of X-rays with gene mutation and then to linear dose response modeling for risk assessment without convincing evidence. This critical belief came to dominate the field, misdirecting radiation genetics, environmental mutagenesis and cancer risk assessment until finally shown to be incorrect decades later [2, 3, 5–7]. Therefore, the failure to receive peer-review as a result of circumventing normal publication procedures at *Science* and publication of his critical data paper in a non-peer reviewed conference proceeding allowed Muller to side-step normal protocols by which the scientific community polices itself, to weed out inferior manuscripts and to enhance the quality of those deemed acceptable. These procedures not only aid society, they are intended to educate the researcher. Muller’s actions were clearly manipulative and self-serving within a context of high stress and excitement. Further, his actions were unfair to the other competitors chasing the gene mutation prize and harmful to society that needs scientists to follow the procedure, even though the entire review process can be frustrating, time-consuming, and very imperfect.

The issue of peer review is relevant to Muller’s Nobel Prize research activities. The question may be raised as to what was the nature of the peer review in the early decades of the twentieth century. While the history of peer in the early part of the twentieth century is not particularly clear due to inadequate record keeping, various biological/medical journals of the early twentieth century conducted their peer-review via a committee/editorial board system, which progressively expanded to include reviewers outside the Editorial Board. The *British Medical Journal* reports a modern sounding peer review process as early as 1893 [1]. A similar set of procedures was described for the journal *Surgery, Gynecology and Obstetrics* in 1905 [1].

An insight into how radiation geneticists viewed peer-review may be gained by a consideration of the actions/leadership of Stewart Gager and the activities of the *American Journal of Botany* (AJB). Gager, who was the co-author of the first paper to show gene mutation as noted above, was on the editorial board of the AJB starting in 1915, being its business manager for 21 years (until 1936). During this time, the journal developed a modern peer-review process with the goal of providing “a hopeful means of preventing publication of inferior papers and improving the quality of others”. This process was in place by 1933. Of significance is that the AJB editorial board had representatives from a broad spectrum of affiliated professional societies, including the American Phytopathological Society, the Ecological Society of America, The American Society of Plant Physiologists, The Mycological Society of America and the Genetics Society of America. Referees were typically selected by an editorial board member with the closest subject matter expertise to the paper. Rejection of a paper had to be approved by at least one member of the board along with the editor-in-chief [23]. This historical reconstruction indicates that peer-review was well developed, sophisticated and built into the core framework of a broad spectrum of the biological sciences in the United States (U.S.) by the early 1930s.

The earliest historical linkage of peer-review to Muller may be seen with the *Journal of Experimental Zoology*. This journal was created in 1903 with the first issue published in 1904. Amongst the founding and longstanding editorial board members was Thomas H. Morgan, who would become Muller’s Ph.D. advisor a decade later. In a retrospective recounting of the journal, the managing editor Ross G. Harrison [13] indicated that peer-review was incorporated from the onset. The paper specifically reported on the percentage of submitted manuscripts rejected over the course of the first 40 years of the journal. In the case of Muller, a search of the Web of Science indicates he published three genetics papers within the *Journal of Experimental Zoology* [15–17]. This information provides unequivocal evidence that Muller was part of a scientific culture of peer review, with an advisor who was a leader on this issue and that Muller personally experienced the peer review process in this journal. Other journals that Muller published in also had established peer-review processes such as *Genetics*, which T.H. Morgan also helped create in 1916 [11], with Muller publishing in this journal five times by 1928. There is also a documented incident of Muller resigning from the editorial board of *Advances in Genetics* over a dispute with the editor M. Dermerc. In this case, Muller was angry because a manuscript was published as a result of a favorable peer-review. Muller was frustrated that Demerec had not selected him as a reviewer. In their exchange of letters, Demerec ended up accusing Muller of attempting to impose his version of censorship [10]. There was, therefore a “culture” of peer-review, that existed and that Muller was part of it from his earliest days as a graduate student at Columbia. These examples are relevant to the Muller story, demonstrating peer-review activity before and within the time period of Muller’s significant work, linking it to the genetics community, to his research mentor T.H. Morgan, one of his rivals, Stewart Gager, and his own published research.

With respect to Muller, it was clear that he knew where his gene mutation interpretational weakness lay. In fact, the gene mutation criticism of Stadler [24, 25] was first brought up by Muller in December, 1927 at the AAAS meeting in Nashville but in a manner that he could control, direct and marginalize. When Stadler [24, 25] raised the issue it was based on new advances in cytogenetics as developed and applied by Barbara McClintock [14]. In fact, it was the Stadler-McClintock criticism that would stick and compel Muller’s research attention for decades as he tried to defend his gene mutation interpretation.

Muller was focused on being the first to produce gene mutation. He knew it was critically important and so did others. The publication actions of Muller (i.e., *Science* journal discussion only and the non-peer-reviewed Congress proceedings) were directed toward being first, even if it meant skirting the protocol of the publication process. Muller also cited the date of publication of his key Proceedings paper incorrectly, making it 1927 rather than 1928. He did this in other subsequent publications, influencing many others to adopt the same publication date error, but all pointing to the notion that Muller was first. In the end, what this meant was that Muller won the race, got the prize and yet would have his key finding, that he produced gene mutations, recognized as incorrect even by his closest colleagues long after his death [9] (See [4] for a detailed review).

Would a professional peer-review had detected the error of Muller’s gene mutation interpretation? While this is not known it is likely that reviewers would have demanded that Muller include a methods section, and a legitimate discussion component to the paper at the least. These would have involved delays. What would have happened in a review process will remain speculative. However, one can see in Muller’s actions how they were not fair to this competitors as well as the scientific community and society who need scientific contributions vetted in an objective manner that ensures an appropriate evaluation of the submitted manuscript.

## Conclusion

This paper reveals that the strategy of Hermann J. Muller to obtain primacy for his discovery of radiation-induced gene mutations led to his avoiding peer-review, publishing a paper that had significant limitations despite its Nobel Prize award quality. Muller’s actions were troubling as he placed his prize-seeking goals ahead of his responsibility to the field and social obligation of playing by the same publication rules as other researchers. The peer-review process is important and even Muller’s Nobel Prize winning research would have profited from this. Furthermore, we have now learned with modern nucleotide assessment technology, that his Nobel Prize winning findings of producing gene mutations by high doses of X-rays were not gene mutations, but gross gene deletions and other major chromosome rearrangements, a finding that confirmed the doubts of some leading geneticists of his era who had clearly suspected that he had incorrectly interpreted his findings.

## Abbreviations

AAS: American Association for the Advancement of Science
AJB: American Journal of Botany
NAS: National Academy of Sciences
PNAS: Proceedings of the National Academy of Sciences
U.S.: United States
